# Opposite correlation of 25-hydroxy-vitamin D- and 1,25-dihydroxy-vitamin D-metabolites with gestational age, bone- and lipid-biomarkers in pregnant women

**DOI:** 10.1038/s41598-021-81452-9

**Published:** 2021-01-21

**Authors:** Oleg Tsuprykov, Saban Elitok, Claudia Buse, Chang Chu, Bernhard Karl Krämer, Berthold Hocher

**Affiliations:** 1 Institute for Laboratory Medicine, IFLB, Berlin, Germany; 2 Fifth Department of Medicine (Nephrology/Endocrinology/Rheumatology), University Medical Centre Mannheim, University of Heidelberg, Heidelberg, Germany; 3 LADR GmbH, MVZ Berlin, Berlin, Germany; 4 Key Laboratory of Study and Discovery of Small Targeted Molecules of Hunan Province, Department of Pharmacy, School of Medicine, Hunan Normal University, Changsha, Hunan China; 5 Reproductive and Genetic Hospital of CITIC-Xiangya, Changsha, China; 6 Institute of Medical Diagnostics, IMD Berlin, Berlin, Germany

**Keywords:** Biochemistry, Hormones, Steroid hormones

## Abstract

25-Hydroxyvitamin D (25OHD) and 1,25-dihydroxyvitamin D (1,25(OH)_2_D) need to be bound to carrier proteins to be transported to their target cells. The majority of either 25OHD or 1,25(OH)_2_D is bound to vitamin D-binding protein (DBP), a smaller fraction is bound to albumin and only very small amounts of 25OHD or 1,25(OH)_2_D are free. Albumin-bound 25OHD or 1,25(OH)_2_D is relatively easily available after dissociation from albumin. Thus, the sum of free and albumin-bound forms is called bioavailable 25OHD and bioavailable 1,25(OH)_2_D. Total 25OHD and 1,25(OH)_2_D are defined as the sum of free, albumin-bound and DBP-bound 25OHD and 1,25(OH)_2_D, respectively. This cross-sectional study in 427 pregnant women compared the correlation of the six vitamin D compounds with biomarkers of bone health, lipid metabolism, kidney function, endocrine parameters, and group B water-soluble vitamins. Among the 25OHD metabolites analysed, total 1,25(OH)_2_D showed clearly the best correlation with calcium, bone-specific alkaline phosphatase, adiponectin, LDL, HDL, urea, thyroxine, and group B water-soluble vitamins. When comparing the three 25OHD metabolites, both free 25OHD and bioavailable 25OHD showed overall good correlations with calcium, bone-specific alkaline phosphatase, adiponectin, LDL, HDL, urea, thyroxine, triiodothyronine, and group B water-soluble vitamins, The correlations of 1,25(OH)_2_D and 25OHD metabolites went always in opposite directions. Only PTH correlates always inversely with all six vitamin D compounds. In conclusion, free 25(OH)D and bioavailable 25(OH)D are more precise determinants of the vitamin D status than total 25(OH)D in normal pregnancy, whereas total 1,25(OH)_2_D is superior to free and bioavailable 1,25(OH)_2_D. Except for PTH, correlations of 25(OH)D and 1,25(OH)_2_D metabolites with typical clinical chemistry readouts go in opposite directions.

## Introduction

Both 25OHD and 1,25(OH)_2_D, being lipophilic molecules, have a special carrying protein, enabling their transport in the circulation, protection from hepatic degradation and accumulation of circulating reserves, called vitamin D-binding protein (DBP). In the serum, 85–90% of the whole 25OHD/1,25(OH)_2_D pool is present in the DBP-bound condition. The remaining 10–15% are loosely bound to serum albumin and lipoproteins (10–15%), and just a tiny fraction of 25OHD/1,25(OH)_2_D (less than 0.1% of 25OHD and about 0.4% of 1,25(OH)_2_D^1,2^) is available in the free, unbound condition. Thus, from this point of view, the terminology “total 25OHD” “total 1,25(OH)_2_D”, which will be used throughout this manuscript, refers to a sum of three 25OHD/1,25(OH)_2_D fractions: DBP-bound, albumin-bound (the latter fraction is also called bioavailable, since this binding is very weak and spontaneous dissociation of vitamin D metabolites from albumin is very easy to occur), and free compounds. Since serum levels of liver-produced DBP is subject to substantiate changes under numerous physiologic and pathologic conditions (genetic background, endocrine status in particular with regards to estrogens, liver diseases and kidney diseases), the values of total 25OHD—the current gold standard vitamin D status marker—might not precisely clarify the status of vitamin D, particularly in such conditions as normal pregnancy, estrogen-containing oral contraceptive intake, as well as liver and renal pathologies.

Since the mid-1980s it is known that free 25OHD/1,25(OH)_2_D determination is possible based on the laws of protein–ligand binding kinetics using a formula employing serum concentrations of total 25OHD, DBP, albumin, as well as 25OHD/1,25(OH)_2_D-DBP and 25OHD/1,25(OH)_2_D-albumin binding affinity constants, which was originally suggested by Bikle et al.^3^.

The present study was designed to perform for the first time a systematic head-to-head comparison of three ways of measuring 25-hydroxyvitamin D (free, bioactive, ant total 25OHD) with three ways to measure 1,25-dihydroxyvitamin D (free, bioactive, ant total 1,25(OH)_2_D) using pregnant women as a test system, because vitamin D binding protein is incresed in this population.

## Materials and methods

### Study population

The study was a part of the Berlin Birth Cohort (BBC) study^4,5^. This study was approved by the local ethical committee of the Charité—Universitätsmedizin Berlin, Campus Mitte, Berlin, Germany and written informed consent was obtained from all participants. The study cohort consisted of 427 Caucasian pregnant healthy women aged > 18 years. All methods were carried out in accordance with relevant German guidelines and regulations.

The study participants were categorized into three trimester groups according to their gestational age: the 1st trimester (≤ 12 weeks + 6 days), the 2nd trimester (13 weeks–27 weeks + 6 days), and the 3rd trimester (≥ 28 weeks until delivery). The mean gestational age of the entire study cohort was 18.49 weeks (n = 427). The group sizes were 211, 78, and 135 participants in 1st, 2nd, and 3rd pregnancy trimesters, respectively.

The study was carried out at clinical laboratory medical institution in Berlin, Germany. Venous blood samples were collected in the period from May till June 2016. The study was approved by the local ethics committee.

### Measurement of 25OHD and 1,25(OH)_2_D metabolites, DBP, and albumin

All analytes were measured in serum samples. Total 25OHD (hereafter “25OHD” is referred to as a sum of 25OHD_2_ and 25OHD_3_ metabolites) was measured by Abbott Architect i2000 (Abbott Laboratories, Wiesbaden, Germany) using Abbott Architect 25OHD automated chemiluminescent microparticle immunoassay (Abbott Laboratories, Wiesbaden, Germany). As declared by the assay manufacturer, the kit has the following cross-reactivities with vitamin D metabolites: 98.6–101.1% with 25OHD_3_, 54.0% with 25OHD_2_, 101.9–189.2% with 24,25OH_2_D_3_, 71.4–114.2% with 24,25OH_2_D_2_, and < 1% with other vitamin D forms (vitamin D_3_, vitamin D_2_, 1,25(OH)_2_D_3_, and 1,25(OH)_2_D_2_). Total 1,25(OH)_2_D (hereafter “1,25(OH)_2_D” is referred to as a sum of 1,25(OH)_2_D_2_ and 1,25(OH)_2_D_3_ metabolites) was determined using IDS-iSYS 1,25 VitD^XP^ chemiluminescence-immunoassay on the IDS-iSYS Multi-Discipline Automated system (both by IDS Immunodiagnostic Systems GmbH, Frankfurt am Main, Germany). According to the manufacturer’s data sheet, this assay has the following cross-reactivities with 1,25(OH)_2_D metabolites: 97% with 1,25(OH)_2_D_3_, 72% with 1,25(OH)_2_D_2_, 106.7% with 1,24,25OH_3_D_3_, and < 1% with other vitamin D metabolites. Serum DBP was determined by a polyclonal antibody immunoassay (Immundiagnostik AG, Bensheim, Germany). Serum albumin was assessed using an automated analyzer (Beckman Coulter Inc., Krefeld, Germany). Regarding the quality control in total 25OHD, total 1,25(OH)_2_D and albumin measurement, our lab constantly fulfill the requirements of the round robin tests organized by the Reference Institute for Bioanalytics (RfB)—one of the two German proficiency testing organizations which have been officially charged by the German Medical Association, in which our lab participates regularly. Regarding DBP, this analyte does not belong to the list of routine clinical biomarkers and was measured exclusively for the present study. However, this kit is IVD- (“In-Vitro-Diagnostic Medical Device”) and CE- (“*Conformité Européene”*) certified, which means that it can be used in-vitro for the examination of specimens, including blood- and tissue donations, derived from the human body within the European Economic Area.

Free 25OHD was measured based on the laws of protein–ligand binding kinetics by calculation method using a formula employing serum concentrations of total 25OHD, DBP, albumin, as well as 25OHD-DBP and 25OHD-albumin binding affinity constants, which was originaly suggested by Bikle et al.^6^:

$$Free \;25\left( {{\text{OH}}} \right){\text{D }} = \frac{{Total \;25\left( {{\text{OH}}} \right){\text{D }}}}{{1 + K_{alb} \times albumin + K_{DBP} \times DBP}}$$

where Free 25OHD = concentration of free 25OHD in mol/L; K_alb_ = affinity constant between 25OHD and albumin equal to 6 × 10^5^ M^−1^; K_DBP_ = affinity constant between 25OHD and DBP, equal to 7 × 10^8^ M^−1^^6,7^; albumin = concentration of total serum albumin in mol/L; DBP = concentration of total vitamin D-binding protein in mol/L; Total 25OHD = concentration of total 25OHD in mol/L.

Utilizing exactly the same principle, but using 1,25(OH)_2_D-specific binding affinity constants—K_DBP_ = 4.0 × 10^7^ M^−1^ and K_alb_ = 5.4 × 10^4^ M^−1^—we calculated free 1,25(OH)_2_D^6,7^:

$$Free 1,\;25\left( {OH} \right)_{2} D{ } = \frac{{Total\; 1,25\left( {OH} \right)_{2} D }}{{1 + K_{alb} \; \times \;albumin + K_{DBP} \; \times \;DBP}}$$

Bioavailable 25OHD and bioavailable 1,25(OH)_2_D were calculated using the following formula based on precalculated free 25OHD or free 1,25(OH)_2_D levels, respectively^8,9^:

$$Bioavailable\;D = Free \, D + albumin - bound\;D = Free \, D + K_{alb} \; \times \; \, albumin\; \times \; \, Free \, D$$

where D = concentration of vitamin D metabolite (25OHD or 1,25(OH)_2_D) in mol/L; K_alb_ = affinity constant between a respective vitamin D metabolite (6 × 10^5^ M^−1^ for 25OHD and 5.4 × 10^4^ M^−1^ for 1,25(OH)_2_D) and albumin in mol^−1^; albumin = concentration of serum albumin in mol/L.

### Measurement of other serum analytes

The detailed data on diagnostic assays and instrumental devices used for measurement of other serum parameters (calcium, PTH, bone-specific alkaline phosphatase, LDL, HDL, hemoglobin, etc.) are provided in Supplementary Table 1.

**Table 1:** Biochemical characteristics of the study population. Mean values ± standard deviation of age and gestational age, vitamin D metabolites and biochemical, hematological, and endocrinological serum parameters in 1st, 2nd, and 3rd pregnancy trimesters.

Variable, units	1st trimester of pregnancy, mean ± SD	2nd trimester of pregnancy, mean ± SD	3rd trimester of pregnancy, mean ± SD	Changes throughout pregnancy
T25OHD, ng/mL	18.80 ± 9.96	20.26 ± 11.09	20.55 ± 11.56	↔
F25OHD, pg/mL	2.84 ± 1.54	2.41 ± 1.41	2.31 ± 1.48***	↓
B25OHD, ng/mL	1.05 ± 0.57	0.76 ± 0.47***	0.69 ± 0.44***	↓
T1,25(OH)_2_D, pg/mL	105.80 ± 42.59	150.95 ± 47.28***	163.28 ± 43.68***	↑
F1,25(OH)_2_D, pg/mL	0.27 ± 0.10	0.30 ± 0.10**	0.31 ± 0.10***	↑
B1,25(OH)_2_D, pg/mL	9.05 ± 3.47	8.84 ± 2.93	8.58 ± 2.68	↔
Age, years	30.63 ± 6.36	29.54 ± 5.28	29.87 ± 5.86	↔
Gestational age, weeks	7.98 ± 1.84	21.29 ± 4.06***	33.25 ± 2.19^§§§^, ***	↑
PTH, pg/mL	15.37 ± 10.76	14.60 ± 13.48	17.93 ± 13.98	↔
BSAP, µg/mL	12.93 ± 5.12	13.97 ± 5.86	18.01 ± 8.21^§§§,^***	↑
Calcium, mmol/L	2.38 ± 0.12	2.25 ± 0.13***	2.24 ± 0.10***	↓
Phosphate, U/L	4.39 ± 1.65	4.07 ± 1.24	4.33 ± 2.23	↔
DBP, mg/L	532.56 ± 138.73	690.00 ± 171.15***	762.70 ± 254.08***	↑
LDL, mg/dL	118.04 ± 34.00	158.52 ± 39.12***	184.16 ± 40.60^§§,^***	↑
HDL, mg/dL	59.36 ± 13.25	65.10 ± 12.40**	67.48 ± 12.64***	↑
LDL/HDL ratio	2.05 ± 0.61	2.49 ± 0.63***	2.83 ± 0.82***	↑
Adiponectin, µg/mL	9.28 ± 4.15	8.06 ± 3.73*	8.41 ± 3.97	↓
Free T3, pg/mL	3.10 ± 0.38	3.15 ± 0.38	3.03 ± 0.38^§^	↓
Free T4, ng/dL	1.02 ± 0.14	0.87 ± 0.12***	0.85 ± 0.11***	↓
TSH, uU/mL	1.48 ± 0.97	1.54 ± 0.79	1.45 ± 0.66	↔
Hemoglobin, g/dL	12.55 ± 0.94	11.53 ± 0.83***	11.66 ± 0.93***	↓
RBC count, 10^6^/µL	4.34 ± 0.39	3.90 ± 0.35***	4.00 ± 0.34***	↓
WBC count, 10^3^/µL	8.54 ± 2.14	9.55 ± 2.83*	9.75 ± 2.33***	↑
Platelet count, 10^3^/µL	232.55 ± 50.51	214.12 ± 42.94	220.68 ± 54.10	↔
MCV, fL	89.13 ± 5.42	89.77 ± 5.67	89.34 ± 5.39	↔
MCH, pg	29.18 ± 1.96	29.73 ± 2.12	29.32 ± 1.95	↔
MCHC, g/dL	32.75 ± 0.97	33.15 ± 0.96*	32.93 ± 1.11	↔
RDW, %	13.97 ± 1.23	13.99 ± 1.11	13.87 ± 1.16	↔
Hematocrit, %	38.57 ± 2.85	34.86 ± 2.39***	35.51 ± 2.96***	↓
Urea, mg/dL	23.07 ± 5.85	17.97 ± 4.89***	17.93 ± 4.58***	↓
Albumin, g/dL	4.09 ± 0.38	3.45 ± 0.30***	3.30 ± 0.24***	↓
Vitamin B6, ng/mL	14.43 ± 18.70	7.34 ± 7.78***	5.55 ± 13.11^§,^***	↓
Vitamin B12, pg/mL	368.63 ± 157.81	279.23 ± 123.68***	275.75 ± 121.83***	↓
Sodium, mmol/L	137.80 ± 4.46	138.03 ± 2.80	137.77 ± 3.43	↔
Zinc, µmol/L	12.50 ± 2.83	10.13 ± 2.21**	9.86 ± 2.13***	↓

B25OHD, bioavailable 25OHD; B1,25(OH)_2_D, bioavaialble 1,25(OH)_2_D; BSAP, bone-specific alkaline phosphatase; F25OHD, free 25OHD; F1,25(OH)_2_D, free 1,25(OH)_2_D; fT3, free triiodothyronine; fT4, free thyroxine; HDL, high-density lipoprotein cholesterol; LDL, low-density lipoprotein cholesterol; MCH, mean corpuscular hemoglobin; MCHC, mean corpuscular hemoglobin concentration; MCV, mean corpuscular volume of red blood cells; PTH, parathyroid hormone; RBC, red blood cell; RDW, red blood cell distribution width; T25OHD, total 25OHD; T1,25(OH)_2_D, total 1,25(OH)_2_D; WBC, white blood cell. *p < 0.05 versus 1st trimester; **p < 0.01 versus 1st trimester; ***p < 0.001 versus 1st trimester; ^§§^*p* < 0.01 versus 2nd trimester; ^§§§^*p* < 0.001 versus 2nd trimester.

### Statistical analyses

Statistical analyses were performed using SPSS 20.0 (IBM, New York, NY, USA) and GraphPad Prism version 5 software (GraphPad Software, San Diego, CA, USA) as recently described^10,11^. All relevant parameters were tested for normality using a D’Agostino and Pearson omnibus normality test and were found to be not normally distributed (*p* < 0.05) in the majority of the cases. For this reason, we used Spearman’s rank correlation analysis to assess bivariate associations between vitamin D metabolites with each other and with other analytes. The correlation strength (measured as Spearman’s rho values) was classified as follows: ≤ 0.1 no correlation; 0.1–0.3 weak to modest; 0.3–0.49 moderate; > 0.5 strong correlation. To assess statistical differences among three trimester groups we used either one-way ANOVA followed by Tukey post hoc test (in cases when all three trimester groups were normally distributed, as determined by D’Agostino and Pearson omnibus normality text), or Kruskal–Wallis analysis with Dunn’s multiple comparison test (in cases when at least one group out of three analyzed groups was found to be not normally distributed). In both correlation and group comparison analyses, correlations/group differences were considered significant if *p* values were < 0.05.

## Results

### Study participant characteristics

The mean values of participant’s age and gestational age, as well as selected serum biomarkers for the pregnancy overall and for early, mid-, and late pregnancy are presented in Table 1.

### Changes in serum parameters throughout pregnancy

The data on changes of biochemical and biochemical parameters throughout pregnancy (split by three trimesters) are presented in Table 1. Importantly, a progressive increase in DBP during pregnancy was observed (43% increase in 3rd trimester compared to the 1st trimester, *p* < 0.001, Table 1).

Regarding 25(OH)D metabolites, we found no significant changes in total 25OHD throughout pregnancy. In contrast, free 25OHD and bioavailable 25OHD were both significantly decreased with gestational age (Table 1). The dynamics in changes of 1,25(OH)_2_D isoform levels was totally different compared to the behavior of 25OHD compounds. Indeed, total 1,25(OH)_2_D showed a progressive increase throughout pregnancy. The same trend revealed free 1,25(OH)_2_D with however weaker numerical rise. In contrast, bioavailable 1,25(OH)_2_D showed no significant differences between early and late gestation groups (Table 1).

### Associations between 25OHD and 1,25(OH)_2_D metabolites

In general, absolutely all analyzed compounds of 25OHD and 1,25(OH)_2_D showed a significant positive correlation with each other, however, the degree of such association (characterized by Spearman’s rho) varied from 0.15 until 0.98 (*p* < 0.001 for all pairs of comparison, except of the pair total 1,25(OH)_2_D versus bioavailable 25OHD with *p* = 0.002, Table 2). Regarding 25OHD isoforms, total 25OHD, free 25OHD, and bioavailable 25OHD revealed very strong correlation between each other (0.813 < rho < 0.976, Table 2). Regarding interrelations between 1,25(OH)_2_D compounds, the correlation was substantially weaker (0.588 < rho < 0.923, *p* < 0.001 for all pairs of comparison) compared to the ones between 25OHD isoforms and more variable depending on certain pair of comparison. In addition, total 25OHD showed a medium correlation with all three 1,25(OH)_2_D compounds of nearly equal strength (rho around 0.55). In contrast, free 25OHD and bioavailable 25OHD showed much weaked correlation with total 1,25(OH)_2_D than with free 1,25(OH)_2_D and bioavailable 1,25(OH)_2_D (rho = 0.29, 0.55, and 0.63, respectively, see Table 3). The relationship between bioavailable 25OHD and 1,25(OH)_2_D compounds was very similar to interrelations between free 25OHD and 1,25(OH)_2_D.

**Table 2:**
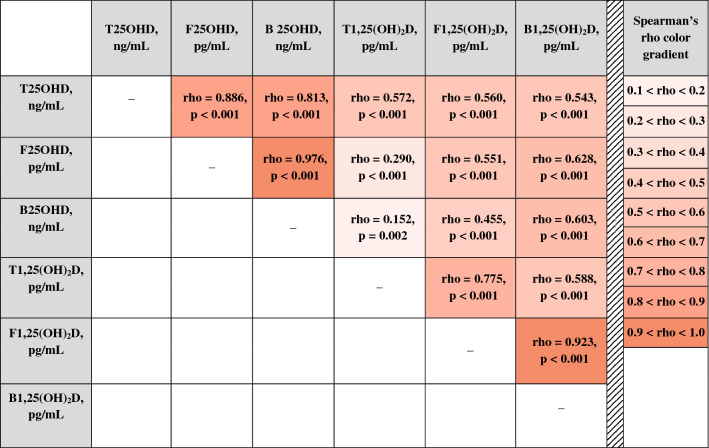
Interrelations between 25OHD and 1,25(OH)_2_D isoforms.

The correlation strength is defined by Spearman's rank correlation coefficient rho. Abbreviations: B25OHD, bioavailable 25OHD; B1,25(OH)_2_D, bioavaialble 1,25(OH)_2_D; F25OHD, free 25OHD; F1,25(OH)_2_D, free 1,25(OH)_2_D; T25OHD, total 25OHD; T1,25(OH)_2_D, total 1,25(OH)_2_D.

### Correlations of free and total 25(OH)D with serum biochemical and hematological parameters

Overall, free 25OHD and, particularly bioavailable 25OHD, showed stronger associations with distinct parameters than total 25OHD (Table 3).

**Table 3:**
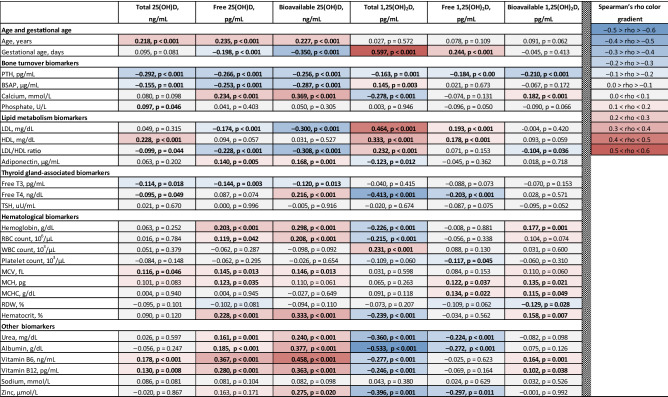
Interrelations between 25OHD and 1,25(OH)and 1,25(OH)_2_D isoforms with age, gestational age, biochemical, hematological, and endocrinological serum parameters.

The correlation strength is defined by Spearman's rank correlation coefficient rho (appears first in each cell). Abbreviations: B25OHD, bioavailable 25OHD; B1,25(OH)_2_D, bioavaialble 1,25(OH)_2_D; BSAP, bone-specific alkaline phosphatase; F25OHD, free 25OHD; F1,25(OH)_2_D, free 1,25(OH)_2_D; fT3, free triiodothyronine; fT4, free thyroxine; HDL, high-density lipoprotein cholesterol; LDL, low-density lipoprotein cholesterol; MCH, mean corpuscular hemoglobin; MCHC, mean corpuscular hemoglobin concentration; MCV, mean corpuscular volume of red blood cells; PTH, parathyroid hormone; RBC, red blood cell; RDW, red blood cell distribution width; T25OHD, total 25OHD; T1,25(OH)_2_D, total 1,25(OH)_2_D; WBC, white blood cell.

Total 25OHD showed no correlation with serum calcium—a key parameter of bone health (rho = 0.080, *p* = 0.098), whereas free 25OHD revealed weak but significant positive correlation with calcium (rho = 0.234, *p* < 0.001, Table 3). Bioavailable 25OHD showed even stronger association with calcium (rho = 0.369, *p* < 0.001, Table 3).

Total 25(OH)D showed no correlation with serum albumin, free thyroxin, urea, adiponectin, LDL, LDL/HDL ratio, vitamin B12, hemoglobin, white blood cell count, and red blood cell distribution width, in contrast to free 25(OH)D, which did show weak but significant correlations with these analytes (Table 3). In addition, despite the presence of the correlation between total 25(OH)D and bone-specific alkaline phosphatase and vitamin B6, it was borderline significant, in contrast to free 25(OH)D, which was correlated with these parameters with *p* < 0.001. The associations of total and free 25(OH)D with other biochemical and hematological parameters are presented in Table 3.

## Discussion

### Summary of the key findings

To the best of our knowledge, the present cross-sectional study is the first report comparing the behavior of the six vitamin D compounds—free, bioavailable, and total 25-hydroxyvitamin D as well as free, bioavailable, and total 1,25-dihydroxyvitamin D—in a relatively large population of healthy pregnant women (n = 427). Among the 1,25-dihydroxyvitamin D metabolites analyzed, total 1,25(OH)_2_D showed clearly the best correlation with components of the endocrine vitamin D system and/or potential cellular targets of activation of the vitamin D receptor (see Table 3). When comparing the three 25OHD metabolites, both free 25OHD and bioavailable 25OHD showed overall good correlations with the analyzed clinical laboratory parameters, bioavailable 25OHD might perform even better then free 25OHD. The only exception from the above described overall findings is PTH. It correlates well with total 25OHD most likely because the parathyroid cells have a highly specific uptake mechanism for bound 25OHD—megalin/cubilin-uptake system, see below. With the exception of PTH, associations of 25(OH)D metabolites and 1,25(OH)_2_D metabolites with typical clinical chemistry readouts goes in opposite directions.

Moreover, our study confirmed the finding of the study by Bouillon et al.^12^ that serum total 25OHD doesn’t markedly change during pregnancy, whereas serum total 1,25(OH)_2_D increased significantly along with serum DBP, which in turn resulted in the increase in free vitamin D metabolites: free 25OHD was declined and free 1,25(OH)_2_D was slightly elevated as total 1,25(OH)_2_D was increased slighthy more than DBP.

### Free 25OHD and bioavailable 25OHD were found to be superior to traditional total 25OHD

Our data suggest that in normal human pregnancy both free 25OHD and bioavailable 25OHD (the latter is tendentially slightly superior to free 25OHD) appear to be more precise indicators of vitamin D status during pregnancy than routine total 25OHD in terms of overall better correlations with gestational age, calcium, bone-specific alkaline phosphatase, adiponectin, hemoglobin and red blood cell count, vitamins B6 and B12 and other analytes (Table 3). In other words, our data are in favor of clinical relevance of free hormone hypothesis for 25OHD.

To date, the data on changes in free/bioavailable 25OHD with total 25OHD throughout pregnancy were analyzed in nine studies^9–11,13–18^, including our own^10,11^. Overall, the data regarding the comparison of the diagnostic potential of free/bioavailable 25OHD versus total 25OHD, reported by these authors, are conflicting due to low sample sizes (varying between 10 and 60 participants), with the exception of two studies by Powe et al.^18^ and Gustafsson et al.^17^ , which was based on 170 and 855 pregnant women, respectively. This discrepancy was discussed in details in our recent publications^11,19^. This study by Powe et al.^18^ differs from ours: just three-fourth of the participating women were healthy and only around 60% were of White ethnicity. Although F25OHD was assessed by calculation method using vitamin D-binding protein (DBP) levels like in our study, DBP in this study was measured using a monoclonal antibody-based ELISA. This DBP measurement technique is considered unreliable, especially when having populations with different ethnic backgrounds due to DBP polymorphism^20–23^. Our study is larger, conducted in an ethnically homogenous cohort and by using the state-of-the-art way to measure DBP. We thus believe that our data are more relevant. Moreover, these results are in good agreement with the findings of our previous study^11^, where we compared total 25OHD with directly measured free 25OHD. Regarding the large longitudinal study by Gustafsson et al.^17^, since the authors did not present the data of bivariate correlation analysis (free 25OHD/total 25OHD versus PTH), a one-to-one comparison of our results with their results is impossible.

### Neither free 1,25(OH)_2_D nor bioavailable 1,25(OH)_2_D showed superiority to total 1,25(OH)_2_D

In contrast to 25OHD—vitamin D’s precursor and reservoir—our data do not support the free hormone hypothesis for 1,25(OH)_2_D—vitamin D’s biologically active metabolite. Indeed, with the exception of PTH, total 1,25(OH)_2_D showed constantly stronger correlations with all analyzed serum biomarkers than free 1,25(OH)_2_D and bioavailable 1,25(OH)_2_D (Table 3).

According to the data of numerous cross-sectional and longitudinal studies, total 1,25(OH)_2_D is known to rise up to threefold versus non-pregnant state^24^. This is the result of the increased production of 1,25(OH)_2_D by 25-hydroxyvitamin D-1α-hydroxylase (CYP27B1) by not only maternal kidneys, but also, although to a lesser extent, by the fetoplacental unit (maternal decidual and fetal trophoblast tissues of the placenta and fetal kidney)^24–26^. We likewise detected total 1,25(OH)_2_D increase throughout gestation. Notably, free 1,25(OH)_2_D but not bioavailable 1,25(OH)_2_D was found to rise throughout pregnancy course. Three other studies^3,17,27^ likewise reported an increase in free 1,25(OH)_2_D with pregnancy course.

### Correlation with PTH stays apart: all six vitamin D isoforms showed nearly equal associations with PTH

PTH—a major secretory product of parathyroid gland and a second, along with 1,25(OH)_2_D, hormonal regulator of calcium homeostasis—was found to be the only biomarker which showed the same direction of correlation (negative correlation) and strength (weak but still significant negative correlation) with all six analysed vitamin D metabolites. The most likely explanation of this finding could be that parathyroid gland, along with kidney and placenta, are exceptional organs, which have megalin/cubilin-mediated system of 25OHD/1,25(OH)_2_D uptake from 25OHD-DBP and 1,25(OH)_2_D-DBP complexes^28,29^ and thus the cells of parathyroid gland are influenced by all circulating isoforms of 25OHD/1,25(OH)_2_D at nearly the equal extent. This mechanism is absent in most of the other cell types and organs, leaving for those cells only the option of the freely circulating 25OHD/1,25(OH)_2_D uptake via passive diffusion through plasma membranes.

### 25OHD and 1,25(OH)_2_D correlate in opposite directions with biochemical and endocrinological biomarkers

The direction of correlation (i.e., positive vs negative) between 25OHD metabolites on one hand, and 1,25(OH)_2_D metabolites on the other hand, with other serum biomarkers was found to be opposite. The most prominent examples are the differences in the correlation directions with such biomarkers as LDL, free thyroxin, urea, vitamins B6, B12, and zinc (Table 3). This emphasizes the different biological roles of total 1,25(OH)_2_D—vitamin D’s fully active isoform, which (total 1,25(OH)_2_D)—is present in the circulation in a picomolar range and have a half-life of 5–80 h^30^ and total 25OHD—vitamin D’s precursor, which is present in the circulation in nearly thousand-fold higher concentration range and have a half-live of 10–40 days^30^ and which is well known to serve as a reservoir/storage fraction of vitamin D, responsible for balancing/minimization of short-time (intraday) vitamin D isoform fluctuations. The 10–100-fold lower affinity between DBP and free 1,25(OH)_2_D compared to the affinity between DBP and free 25OHD (to the lesser extent this is valid likewise for albumin) as well as much higher (up to 100-fold) affinity of nuclear VDR for free 1,25(OH)_2_D than for free 25OHD are factors influencing concentrations of the vitamin D metabolites at the nuclear receptor. Whether or not the strength of the binding of either free 1,25(OH)_2_D or free 25OHD to the nuclear receptor is affecting gene expression in an opposite manner is unknown so far. It is conceivable that hormone receptor ligands with low binding affinity for the receptor may act as partial receptor antagonists, whereas ligands with high affinity fully stimulate the receptor. However, this needs to be proven in adequately designed basic science experiments. In any case, this is the first study reporting differences in correlation strength between 25OHD/1,25(OH)_2_D isoforms versus key clinical parameters. This for sure needs to be repeated in independent cohorts and the underlying molecular mechanisms (see our hypothesis above) need to be explored in future studies.

### Study limitations

The data on free and bioavailable 25OHD and 1,25(OH)_2_D were based on calculations using the affinity constants between 25OHD/1,25(OH)_2_D and DBP and 25OHD/1,25(OH)_2_D and albumin. These affinity constants were measured experimentally in serum samples of non-pregnant individuals and has not been verified in pregnant women so far. However, the possible inaccuracies (a systematic shift in calculated free/bioavailable 25OHD/1,25(OH)_2_D absolute levels *versus* their real values) are not influencing the key findings of our study, because our conclusions depend on the relative changes of free, bioavailable and total 25OHD and 1,25(OH)_2_D, respectively rather than absolute changes of these parameters.

It is very well known that during pregnancy, especially in the 3rd trimester, the blood volume of the pregnant women increases and thus the blood is diluted to a certain extent^31^. However, the influence of hemodilution as a confounding factor in bivariate associations presented in this study will not change the correlation direction. Hemodilution might have just flatten the correlation strength (as presented by Spearman’s rho values), but it will not reverse the direction of correlation.

The accuracy of total 25OHD levels produced by fully-automated commercial immunoassays, especially in the conditions of pregnancy-induced increased DBP levels, is a well-known issue^32^. However, we used the total 25OHD kit with the best diagnostic precision characteristics in pregnancy among the other tested commercial kits when comparing to total 25OHD concentrations measured by mass-spectrometry—a technique considered the gold standard for the measurement of 25OHD^32^. Undoubtedly, the usage of LC–MS/MS might have further improved the data quality.

DBP, being a highly polymorphic protein, was reported to have genotype differences (up to threefold) in affinity constants with 25OHD^33^. Although these data were not confirmed by three other studies^34–36^, we are on the safe side with our key conclusions, because we used quite a monogenic (Caucasian) cohort and we measured DBP with a polyclonal-antibody-based ELISA kit. Finally, we conducted a single-centered study and thus single-centered effects cannot be excluded.

### Conclusions

**Figure 1:**
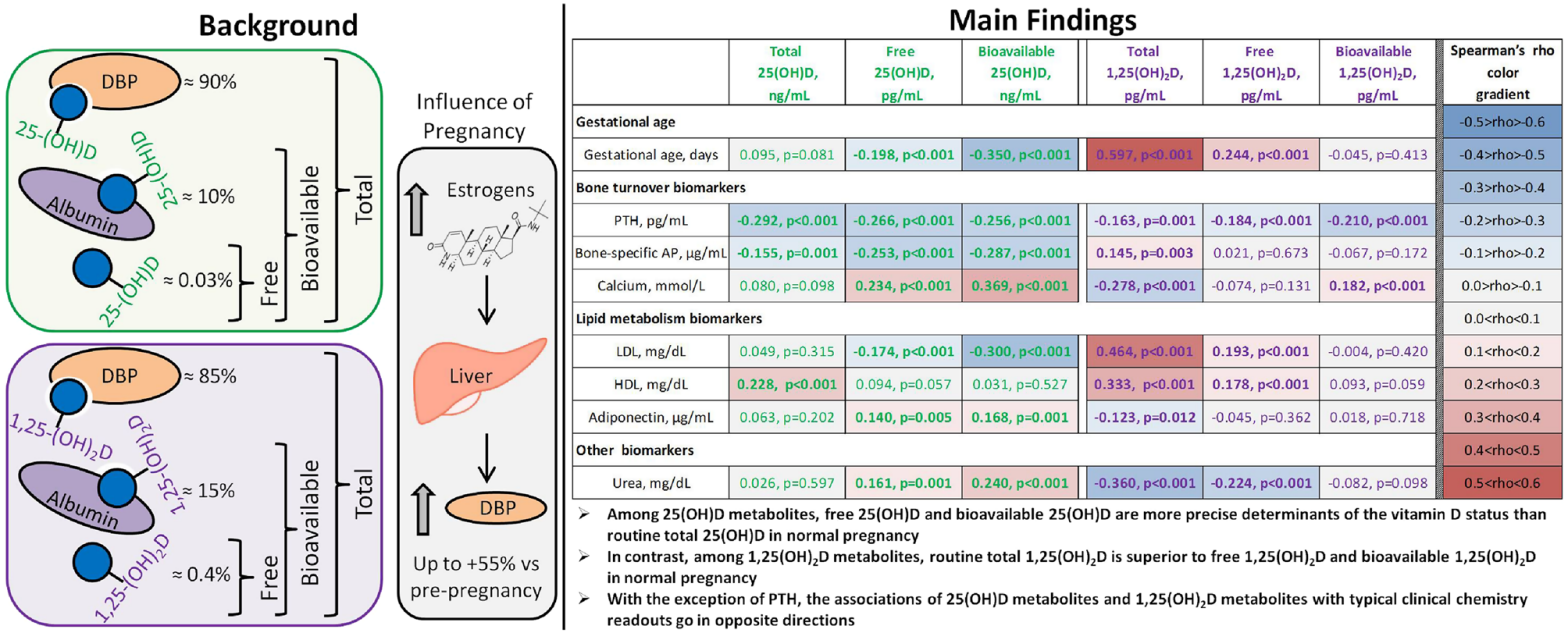
Key findings of our study are: (i) Both free 25(OH)D and bioavailable 25(OH)D showed better correlations with gestational age, bone and lipid metabolism biomarkers than total 25(OH)D. (ii) Total 1,25(OH)_2_D is superior to free 1,25(OH)_2_D and bioavailable 1,25(OH)_2_D. (iii) 25(OH)D metabolites and 1,25(OH)_2_D metabolites correlated with gestational age, bone and lipid biomarkers in opposite directions.

The present study compared the performance of the six vitamin D compounds—free, bioavailable, and total 25-hydroxyvitamin D (free 25OHD, bioavailable 25OHD, total 25OHD) as well as free, bioavailable, and total 1,25-dihydroxyvitamin D (free 1,25(OH)_2_D, bioavailable 1,25(OH)_2_D, total 1,25(OH)_2_D). Among the 1,25-dihydroxyvitamin D metabolites, total 1,25(OH)_2_D clearly showed the best correlation with components of the endocrine vitamin D system and/or potential cellular targets of activation of the vitamin D receptor. When comparing the three 25OHD metabolites, both free 25OHD and bioavailable 25OHD showed an overall good correlation with the analyzed clinical laboratory parameters, bioavailable 25OHD might perform even better then free 25OHD. The only exception is PTH. It correlates well with total 25OHD most likely because the parathyroid cells have a highly specific uptake mechanism for total 25OHD—the megalin/cubilin-uptake system. This specific system acts much faster as compared to the uptake via the cell membrane. With the exception of PTH, correlations of 25(OH)D and 1,25(OH)_2_D metabolites with typical clinical chemistry readouts go in opposite directions suggesting potentially opposite effects of either 25(OH)D or 1,25(OH)_2_D on the nuclear vitamin D receptor in some target cells of vitamin D (see also Fig. 1 summarizing the key findings of our study). This hypothesis based on clinical data needs to be confirmed in adequately designed basic science studies.

## Supplementary Information

Supplementary Information.

## Abbreviations

125(OH)_2_D: 1,25-Dihydroxyvitamin D
25OHD: 25-Hydroxyvitamin D
ANOVA: Analysis of variance
B25OHD: Bioavailable 25-hydroxyvitamin D
B125(OH)_2_D: Bioavailable 1,25-dihydroxyvitamin D
BSAP: Bone-specific alkaline phosphatase
CE: *Conformité Européene*
DBP: Vitamin D-binding protein
ELISA: Enzyme-linked immunosorbent assay
F25OHD: Free 25-hydroxyvitamin D
F125(OH)_2_D: Free 1,25-dihydroxyvitamin D
fT3: Free triiodothyronine
fT4: Free thyroxine
HDL: High-density lipoprotein cholesterol
IVD: In-Vitro-Diagnostic Medical Device
LC–MS/MS: Liquid chromatography–tandem mass spectrometry
LDL: Low-density lipoprotein cholesterol
MCH: Mean corpuscular hemoglobin
MCHC: Mean corpuscular hemoglobin concentration
MCV: Mean corpuscular volume of red blood cells
PTH: Parathyroid hormone
RBC: Red blood cell
RDW: Red blood cell distribution width
SD: Standard deviation
T25OHD: Total 25-hydroxyvitamin D
T125(OH)_2_D: Total 1,25-dihydroxyvitamin D
VDR: Vitamin D receptor
WBC: White blood cell
