# Supplementary material for: Opposite correlation of 25-hydroxy-vitamin D- and 1,25-dihydroxy-vitamin D-metabolites with gestational age, bone- and lipid-biomarkers in pregnant women
Source: Sci Rep. 2021 Jan 21;11:1923. doi: 10.1038/s41598-021-81452-9 (PMC7820257; doi:10.1038/s41598-021-81452-9)
Supplement: Supplementary file 1 — Supplementary Information. [file 41598_2021_81452_MOESM1_ESM.docx]

**Supplementary Table 1. Diagnostic Kits and Analysers Used for the Measurement of**

**Biochemical, Hematological and Endocrinological Parameters in Serum**

| **Parameter** | **Kit, Producent, City, and Country** | **Analyzer, Producent, City, and** |
| --- | --- | --- |
|  | **of Origin** | **Country of Origin** |
|  |  |  |
| Total 25OHD | Architect 25-OH Vitamin D | Abbott Architect i2000sr analyzer, Abbott |
|  | chemiluminescent microparticle | Diagnostics, Wiesbaden, Germany |
|  | immunoassay REF 5P02, Abbott |  |
|  | Diagnostics, Wiesbaden, Germany |  |
|  |  |  |
| Total | IDS-iSYS 1,25 VitD^XP^ assay REF IS-2000, | IDS-iSYS Multi-Discipline Automated |
| 1,25(OH)2D | IDS Immunodiagnostic Systems GmbH, | system, IDS Immunodiagnostic Systems |
|  | Frankfurt am Main, Germany | GmbH, Frankfurt am Main, Germany |
|  |  |  |
| Calcium | OSR61117, Beckman Coulter GmbH, | AU5800 Clinical Chemistry System, |
|  | Krefeld, Germany | Beckman Coulter GmbH, Krefeld, Germany |
|  |  |  |
| Inorganic | OSR6122, Beckman Coulter GmbH, Krefeld, | AU5800 Clinical Chemistry System, |
| Phosphate | Germany | Beckman Coulter GmbH, Krefeld, Germany |
|  |  |  |
| Sodium | ISE, Beckman Coulter GmbH, Krefeld, | AU5800 Clinical Chemistry System, |
|  | Germany | Beckman Coulter GmbH, Krefeld, Germany |
|  |  |  |
| Albumin | OSR6102, Beckman Coulter GmbH, Krefeld, | AU5800 Clinical Chemistry System, |
|  | Germany | Beckman Coulter GmbH, Krefeld, Germany |
|  |  |  |
| Vitamin D- | Vitamin D binding protein ELISA kit with | DSX automated ELISA processing system, |
| binding protein | polyclonal antibodies REF K2314, | Dynex Technologies, Chantilly, VA, USA |
|  | Immundiagnostik AG, Bensheim, Germany |  |
|  |  |  |
| Vitamin B12 | 7K6135, Abbott Diagnostics, Wiesbaden, | Abbott Architect i2000sr analyzer, Abbott |
|  | Germany | Diagnostics, Wiesbaden, Germany |
|  |  |  |
| TSH0 | 7K6230, Abbott Diagnostics, Wiesbaden, | Abbott Architect i2000sr analyzer, Abbott |
|  | Germany | Diagnostics, Wiesbaden, Germany |
|  |  |  |
| Free thyroxine | 7K6534, Abbott Diagnostics, Wiesbaden, | Abbott Architect i2000sr analyzer, Abbott |
|  | Germany | Diagnostics, Wiesbaden, Germany |
|  |  |  |
| Free | 7K6330, Abbott Diagnostics, Wiesbaden, | Abbott Architect i2000sr analyzer, Abbott |
| triiodothyronine | Germany | Diagnostics, Wiesbaden, Germany |
|  |  |  |
| Bone-specific | IDS-iSYS Ostase BAP Assay REF IS- | IDS-iSYS Multi-Discipline Automated |
|  |  |  |

| alkaline | 2800, Immunodiagnostic Systems GmbH, | system, Immunodiagnostic Systems GmbH, |
| --- | --- | --- |
| phosphatase | Frankfurt am Main, Germany | Frankfurt am Main, Germany |
|  |  |  |
| Intact | IDS-iSYS Intact PTH assay REF IS-3200, | IDS-iSYS Multi-Discipline Automated |
| parathyroid | Immunodiagnostic Systems GmbH, | system, Immunodiagnostic Systems GmbH, |
| hormone | Frankfurt am Main, Germany | Frankfurt am Main, Germany |
|  |  |  |
| HDL- | OSR6587, Beckman Coulter GmbH, Krefeld, | AU5800 Clinical Chemistry System, |
| Cholesterol | Germany | Beckman Coulter GmbH, Krefeld, Germany |
|  |  |  |
| LDL- | OSR6283, Beckman Coulter GmbH, Krefeld, | AU5800 Clinical Chemistry System, |
| Cholesterol | Germany | Beckman Coulter GmbH, Krefeld, Germany |
|  |  |  |
| Total | ELISA assay E09, Mediagnost, Reutlingen, | Multiskan^TM^ FC Microplate Photometer REF |
| adiponectin | Germany | 51119000, Thermo Scientific, Shanghai, |
|  |  | China |
|  |  |  |
| Vitamin B6 | REF 31004, REF 31005, and REF 31006, | HPLC Dionex Ultimate 3000, Thermo |
|  | Chromsystems GmbH, Gräfelfing, Germany | Scientific, Waltham, MA, USA |
|  |  |  |
| Urea | OSR6534, Beckman Coulter GmbH, Krefeld, | AU5800 Clinical Chemistry System, |
|  | Germany | Beckman Coulter GmbH, Krefeld, Germany |
|  |  |  |
| Zinc | - | Atomic absorption spectrophotometer ICE |
|  |  | 3000 Series, Thermo Scientific, Rockford, |
|  |  | IL, USA |
|  |  |  |
| Hemoglobin | - | Beckman Coulter UniCelDxH 800 |
|  |  | analyzer, Beckman Coulter, Brea, CA, USA |
|  |  |  |
| Red blood cell | - | Beckman Coulter UniCel DxH 800 |
| count |  | analyzer, Beckman Coulter, Brea, CA, USA |
|  |  |  |
| White blood | - | Beckman Coulter UniCel DxH 800 |
| cell count |  | analyzer, Beckman Coulter, Brea, CA, USA |
|  |  |  |
| Platelet count | - | Beckman Coulter UniCel DxH 800 |
|  |  | analyzer, Beckman Coulter, Brea, CA, USA |
|  |  |  |
| Hematocrit | - | Beckman Coulter UniCel DxH 800 |
|  |  | analyzer, Beckman Coulter, Brea, CA, USA |
|  |  |  |
